# Revalorization of Melon By-Product to Obtain a Novel Sparkling Fruity-Based Wine

**DOI:** 10.3390/foods12030491

**Published:** 2023-01-20

**Authors:** José Ángel Salas-Millán, Encarna Aguayo, Andrés Conesa-Bueno, Arantxa Aznar

**Affiliations:** 1 Postharvest and Refrigeration Group, Universidad Politécnica de Cartagena (UPCT), 30202 Cartagena, Spain; 2 Food Quality and Health Group, Institute of Plant Biotechnology (UPCT), Campus Muralla del Mar, 30202 Cartagena, Spain; 3 JimboFresh International SLL, C/Mina Buena Suerte, 1, La Unión, 30360 Murcia, Spain; 4 Department of Agronomical Engineering, Institute of Plant Biotechnology, UPCT, Paseo Alfonso XIII, 48, 30203 Cartagena, Spain

**Keywords:** fermentative process, melon, beverage, aroma, sparkling, fruit wine

## Abstract

Fresh melons not meeting cosmetic standards were revaluated into sparkling melon-based wine. Firstly, still melon wine was elaborated and bottled into 750 mL bottles, closed with a crown seal, and stored for 10-weeks at 14 °C. The oenological parameters and polar compounds in must, still wine, and during the sparkling process were evaluated during the experiment. The volatile profile was qualified by GC-MS, and the odor activity value (OAV) and relative odor contribution (ROC) were measured for aroma characterization. Results show that sparkling wine resulted in 12% *v*/*v* ethanol. Certain amino acids contributed to the transformation and increase of volatile compounds via Ehrlich’s pathway: leucine to isoamyl alcohol; valine to iso-butyl alcohol; and phenylalanine to phenethyl alcohol. The volatile compounds also increased after the first fermentation, principally in acetate and ethyl esters, and higher alcohols. Isoamyl acetate, ethyl decanoate, 3,6-nonadienyl acetate, and (E,Z)-nonadien-1-ol had the highest OAV and ROC values among the volatiles; this contributed to the sweet, fruity, banana, tropical, nutty and melon aroma in this sparkling wine. Sensory evaluation (100 to 40) was evaluated according to International Organisation of Vine and Wine compendium, the final product (10-week) scored 92 points, with great visual, nose, and taste values. This study demonstrates how by-products revalorization can provide new products such as this novel sparkling wine with a characteristic and distinctive aroma, good sensory acceptance and market potential.

## 1. Introduction

Food loss refers to any food that is discarded, incinerated, or otherwise disposed of along the food supply chain from harvest/catch/slaughter up to but excluding retail level, and which is not used for any other productive use, such as animal feed or seed [1]. In this context, fresh products can be rejected owing to superficial cosmetic imperfections, color, shape, and size after preparation and packaging. These imposed cosmetic quality standards may lead to food losses with significant environmental impacts (land use, water consumption, greenhouse gas emissions, etc.) and financial implications. However, reducing and preventing food loss and waste can increase food security, foster productivity and economic efficiency, promote resource and energy conservation, and address climate change, which in turn, could also decrease climate change-related shocks to the supply chain [2]. In this context, the agriculture sector must tackle the issue of stimulating a circular economy model which enables superficially cosmetic imperfect fruits and vegetables to be utilized in novel food products such as fruit beverages and fruit-based wines by alcoholic fermentation [3]. This research presents an example of revalorizing melon by-products—melons rejected for failing to meet cosmetic standards—to obtain a novel sparkling melon-based wine.

Grapes are traditionally the most used fruit in wine production, but other examples of fermented beverages can be found, made from rice, honey, and fruits including persimmon and kiwi [4,5,6]. Various fruits are produced in large amounts around the world to produce alcoholic beverages through the fermentation process. The technique is related to traditional winemaking in that it involves alcoholic fermentation using yeast—usually *Saccharomyces cerevisiae*—to produce ethanol, carbon dioxide (CO_2_), and other secondary metabolites that enhance the volatile aroma profile, such as esters and higher alcohols [7]. Some wines are made from fruits other than grapes, such as cider which is made from fermented apples and is one of the most popular types of fruit-based wine [8], and it is commonly used in Europe. Fruits such as strawberries, plums, and peaches are used in the USA and Canada for fruit-based wine, whilst mango and pineapple are used in Asia [9]. Depending on the CO_2_ content in the wine, they are classified into “still” or “sparkling” wines [10]. In sparkling wine production, CO_2_ is generated which produces effervescence due to the use of yeast in a second step after the first alcoholic fermentation. Sparkling wines are frequently classified according to the method of production, the three main approaches are: traditional (*champenoise*), transfer, and bulk (*charmat*). With the traditional method, sparkling wines are produced by two steps of fermentation. After the first fermentation is prepared, the *cuvée*, where a rich saccharose solution and nutrients (*liqueur de tirage*) and yeast is added to start the second fermentation to produce CO_2_ inside sealed bottles [4]. These wines are considered for special occasions due to their additional value, their positive mouthfeel increment, their perception of volatile compounds, and their sweetness in consumers [11]. During this second fermentation, the yeast metabolism affects the aroma composition and the chemical composition which may improve the organoleptic perception [12].

Melon (*Cucumis melo* L.) is cultivated in several parts of the world thanks to its adaptability to many types of soils and temperatures. According to FAOSTAT [13], China was the world’s foremost producer of melon (49% of the total, 14 million tons), followed by Turkey and India, although Spain was the world’s main exporter, producing 600,000 t of which 440,000 t were exported, which accounts for around 20% of total global exportation. There are different *C. melo* botanical varieties with morphological, physiological, and organoleptic profiles, such us *cantalupensis*, *reticulatus*, *inodorus*, *ameri*, *flexuosus*, *chate*, *dudaim*, *tibish*, *acidulus*, *momordica*, *conomon*, *makuwa* and *chinensis* [14]. *C. melo* var. *reticulatus* is the most accepted for its sweetness, pulp texture, and aroma, with highly volatile contents such as esters (butyl acetate, ethyl isobutyrate, hexyl acetate, propyl 2-methyl butanoate, 3-methyl-2-butenyl acetate) that improve its aroma attributes [15]. Previous authors have reported melon-based wines [3,16] and have described the main volatiles in melon distillates [17] but, for the moment, there has been no scientific research focused on the development of a sparkling melon-based wine, from *C. melo* var. *reticulatus*, using fresh melon rejected for the cosmetic reasons mentioned above. These melons cultivar have a huge potential aroma profile for alcoholic fermentation. Thus, the aim of this research is to obtain a sparkling wine using fresh melons with cosmetic imperfections, evaluating the physico-chemical, polar compounds, and aroma changes during the sparkling process and then performing a sensory evaluation at the end of the process.

## 2. Materials and Methods

### 2.1. Pilot-Scale Melon-Based Wine

**Figure 1:**
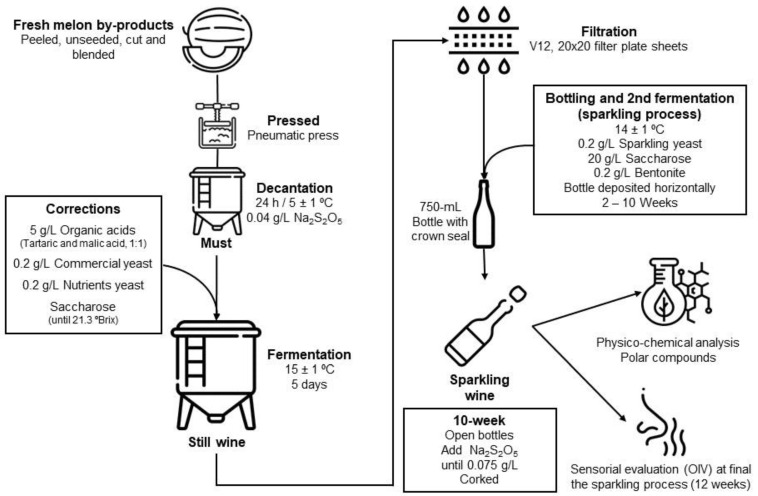
Scheme of the production of melon wine and sparkling wine.

Fresh melon *(Cucumis melo* var. *reticulatus)* Okashi^®^ cultivar was obtained from JimboFresh International Coop. (La Unión, Murcia, Spain). The melons received had failed to meet cosmetic standards, mainly because of their tiny caliber (10–12) and a few sunspots on the skin. Following previous melon-based wine reports [3], we scaled up the process in a winery pilot plant Figure 1, located in the University (Universidad Politécnica de Cartagena, Spain). Fresh melons were hand peeled and cut into four pieces before being blended in a commercial crusher-destemmer (Enoitalia, Florence, Italy) prior to being pressed with a pneumatic press (Bucher Vaslin, Chalonnes-sur-Loire, France) to make the initial must. The must was obtained after decanting for 24 h, at 5 °C adding 0.04 g/L of sodium metabisulfite (Na_2_S_2_O_5_) and enriched with 5 g/L of tartaric and malic acids (1:1, *w/w*), 0.2 g/L of commercial yeast (Zymaflore^®^, Laffort, Bordeaux, France), 0.2 g/L of nutrients yeast (Superstart^®^ Blanc & Rosé, Laffort, Bordeaux, France) and added commercial saccharose until 21.3 °Brix was obtained in the must Figure 1.

After five days at 15 °C, the first fermentation finished obtaining a melon wine named “Still Wine”. Subsequently, that wine was filtered through filter plate sheets (V12, 20 × 20, Gruppo Cordenons SpA, Milano, Italy) with a filter plate (Filtro Jolly 20, MORI, Tavarnelle Val di Pesa, Italy). The sparkling process was based on Martínez-García et al. [12] with slight modifications: adding 0.2 g/L of commercial sparkling yeast Zymaflore^®^ Spark (Laffort, Bordeaux, France) used for the second fermentation in this base melon-based wine and 0.2 g/L of yeast nutrients, and 20 g/L of saccharose, and 0.2 g/L of bentonite were also added. The wine was then bottled in 750 mL bottles closed with a crown seal and stored horizontally in a chamber at 14 °C. At the end of the process (10 weeks), the bottles were placed in a desk and the lees were mixed (*remuage*) and gradually turned and inclined into a vertical position, so the sediment from the lees was deposited in the neck of the bottle and then withdrawn at the end of the process. Sodium metabisulfite was added to reach 0.075 g/L of total SO_2_ and the bottles were corked. The physico-chemical changes and aroma obtained during this second fermentation, sparkling wine, were monitored at six time points of the process (0, 2, 4, 6, 8, and 10 weeks).

### 2.2. Physico-Chemical Analysis

Physico-chemical parameters such as alcohol strength (% *v*/*v*), pressure, total soluble solids (TSS), residual sugar, sugar-free extracts, and total and free sulfide, were measured using the standardized method by OIV [18] in the melon must, the still wine, and during the sparkling process. The pH, total acidity (TA), volatile acidity (VA) and color were determined following the methods from Salas-Millán et al. [3].

The total polyphenol content (TPC) and antioxidant capacities (FRAP and TEAC) were ascertained with a multiscan plate spectrophotometer (Tecan infinite M200, Männedorf, Switzerland after diluting the melon must, still wine, and sparkling wine samples in water (1:5), as per Salas-Millán et al. [19].

### 2.3. Analysis of Polar Compounds

#### 2.3.1. Individual Sugars and Organic Acids

The analysis was performed as per Ortiz-Duarte et al. [20], using ultra-high-performance liquid chromatography (UHPLC) instrument (Shimadzu, Kyoto, Japan) which included a DGU-20 A degasser, LC-170 30AD quaternary pump, SIL-30AC autosampler, CTO-10AS column heater, refractive index detector (RID), and SPDM-20 A diode array detector (DAD). Chromatographic separation was performed at 65 °C with a mobile phase of 2.5 mM H_2_SO_4_ at 0.6 mL/min for 30 min using a Rezex RAM column (300 × 7.8 mm, 8 μm particle size; Phenomenex, Macclesfield, UK). Authentic standards were used to identify and quantify sugars and organic acids (Sigma, St Louis, MO, USA). Calibration curves were created for each standard using at least six data points. A 5 mL must, still wine or sparkling wine aliquot was centrifuged at 14,000× *g* for 15 min at 4 °C, and the supernatant was further purified by solid phase extraction Phenomenex C18-SPE (Torrance, CA, USA) conditioned columns (5 mL of MeOH + 5 mL of water + 5 mL of air). The purified extract was filtered with a polyamide 0.20 μm syringe filter and diluted tenfold prior to analyses. Individual sugars and organic acids were expressed in g/L, except for fumaric acid which was presented in mg/L.

#### 2.3.2. Individual Amino Acids

The separation and analysis of samples was performed with an HPLC/MS system consisting of an Agilent 1290 Infinity II Series HPLC (Agilent Technologies, Santa Clara, CA, USA) equipped with an Automated Multisampler module and a High-Speed Binary Pump and connected to an Agilent 6550 Q-TOF Mass Spectrometer (Agilent Technologies, Santa Clara, CA, USA) using an Agilent Jet Stream Dual electrospray (AJS-Dual ESI) interface. Experimental parameters for HPLC and Q-TOF were set in MassHunter Workstation Data Acquisition software (Agilent Technologies, Santa Clara, CA, USA, Rev. B.10.1.48). Standards with known concentrations of amino acids were prepared in water. Both standards and samples were passed through 0.22 μm filters. Then 20 μL of each standard or sample was injected onto a Zorbax Eclipse Plus C18 HPLC column (100 × 2.1 mm, 1.8 μm,), thermostatted at 40 °C, and eluted at a flow rate of 0.4 mL/min. Chromatographic conditions were run in accordance with Giordano et al. [21] with a slight modification: mobile phase A, consisting of 0.1% TDFHA (tridecafluoroheptanoic acid) (*w/v*) in MilliQ water and mobile phase B, consisting of 0.1% TDFHA (*w/v*) in acetonitrile, were used for the chromatographic separation. The initial HPLC running conditions were solvent A:B 90:10 (*v*/*v*). The gradient elution program was 10% solvent B for 3 min; a linear gradient from 10 to 40% solvent B in 5 min; another linear gradient from 40 to 100% solvent B in 5 min; 2 min at constant 100% solvent B. The column was equilibrated with the starting composition of the mobile phase for 5 min before each analytical run.

The mass spectrometer was operated in the positive mode. The nebulizer gas pressure was set to 40 psi, whilst the drying gas flow was set to 14 L/min at a temperature of 275 °C, and the sheath gas flow was set to 12 L/min at a temperature of 300 °C. The capillary spray, nozzle, fragmentor, and octopole RF Vpp voltages were 3500 V, 100 V, 360 V, and 750 V respectively. Profile data in the 50–400 *m*/*z* range were acquired for MS scans in 2 GHz extended dynamic range mode. A reference mass of 121.0509 was used. Data analysis was performed with MassHunter Qualitative Analysis Navigator software (Agilent Technologies, Santa Clara, CA, USA, Rev. B.80.00). Extracted ion chromatograms of different amino acids, were obtained from their molecular formula Table 1 and were measured in µmol per liter of must, still wine, or sparkling wine (mM).

### 2.4. Analysis of Volatile Compounds by GC-MS

The volatile profiles were extracted from samples using headspace solid-phase micro-extraction (HS-SPME), and identified utilizing gas chromatography (Agilent 7890B) linked to a mass spectrometer (Agilent MSD 5977A) with an autosampler (Gerstel MPS 2XL Twister) according to Salas-Millán et al. [3]. The NIST database provided the mass spectrum and retention index (RI) via the Kovats Index (KI) for comparison in the identification of the volatile substances. RI values were computed employing the same GC-MS settings and an n-alkane external standard solution C8-C20 (Sigma-Aldrich, St. Louis, MO, U.S.A.). As a semi-quantification, the GC peak area ratio of each volatile in the total ion chromatogram to the internal standard peak area was utilized [22], and measured in mg/L of the must, wine (first fermentation), or sparkling wine.

### 2.5. Odor Activity Value (OAV) and Relative Odor Contribution (ROC)

The traditional indicators of odor activity values (OAV) and relative odor contributions (ROC) were employed to quantify the sensory contribution of aromatic compounds to wine flavor [23]. A compound’s concentration divided by its odor threshold value gave the OAV, as stated by other authors [24,25,26,27,28,29,30]. The ratio of the compound’s OAV to overall OAV for each wine is used when calculating the ROC for each aroma component.

### 2.6. Sensory Evaluation

Sensory evaluation was conducted in a normalized tasting room (22 °C) using standardized wine glasses containing 15 mL of melon sparkling wine. The sensory panel was mainly composed of 15 research group judges (eight women and seven men between the ages of 30 and 55), and the sensory evaluation assessment was performed following OIV 332A/2009 resolution [31], in which judges rated several aspects Table 2. The scores for each sensory attribute were written down, with the overall score being produced by adding individual attribute values. This trained sensory evaluation does not require an ethical statement. Before starting this sensory evaluation, the research team explained the scope and details of the project to the participants, including the purpose of the research, the identity of the researchers, information on data protection, privacy, and data retention, the right not to take part (participation was voluntary), the right to withdraw, and contact details for any questions. Finally, participants signed a written consent form, confirming that they had read it and questions had been answered.

### 2.7. Statistical Analysis

A completely randomized design was performed with three replicates in the must and still wine, and per week during the sparkling process. A one-way ANOVA (*p* < 0.01) was carried out to determine the effect of fermentation and time of storage for sparkling wine. Mean values were compared by LSD multiple range test to identify significant differences among samples.

## 3. Results and Discussion

### 3.1. Physico-Chemical Characterization for First Alcoholic Fermentation (Still Wine) and During the Second Fermentation Process (Sparkling Wine)

Table 3 shows the principal physico-chemical parameters of the must, still wine, and sparkling melon wine. After melon must optimization (Section 2.1), once commercial saccharose had been added, the TSS reached 21.3 °Brix and 90.55 g/L of residual sugar (as the sum of total individual sugar, Table 4). The enrichment with tartaric acid and malic acid (5 g/L of 1:1 tartaric and malic acid) provided 6.44 g/L for TA, 0.10 g/L for volatile acidity, and a pH of 4.4. The initial sodium metabisulfite added in the must provided 11.5 mg/L and 25.6 mg/L, free and total SO_2_, respectively, and it avoided prior contamination before alcoholic fermentation. The color of the must was defined as a pale greenness (109 °h) resembling the typical green color of the melon pulp.

After the first alcoholic fermentation, the still wine was obtained and TSS dropped to <9 °Brix and remained stable during the sparkling process without significant differences. The alcoholic grade reached 12.3° and was stable in the sparkling melon process (12.3° to 12.5°). Likewise, residual sugar decreased with fermentation, from 90.55 g/L in the must to 5.72 g/L in the still melon wine, and after the incorporation of 20 g/L of commercial saccharose before the second fermentation, the residual sugar decreased to 5.38–10.23 g/L in the sparkling wine; a decrease in the sugar concentration with the storage time was detected.

Regarding the sugar-free extract, the high value obtained in the must (142 g/L) was due to saccharose added and the presence of suspension pulp, dropping with the fermentation process and the filtration process in the still melon wine (25 g/L) and remaining quite stable during the sparkling wine process (20.6 to 25.1 g/L). Sugar-free extract is an important qualitative parameter for evaluating fullness and harmony in wine, this parameter should usually be below 25 g/L [23], as obtained in the sparkling wine. The pressure reached 2 atm in the second week after second fermentation and remained stable throughout the sparkling process.

TA was maintained in the range of 5.62 to 6.76 g TA/L, without significant differences during the time studied for the sparkling process. As expected, the volatile acidity increased with the first alcoholic fermentation, 0.27 g/L was obtained in the still melon wine, and it ranged from 0.23 to 0.35 g/L during the sparkling process. The pH slightly decreased to 3.9 in the still wine with similar and stable values for the sparkling wines (4.0 to 4.1).

Regarding the color parameters, the first fermentation, induced an increase in the °h (178°) and a slight reduction in L*, with a green color being obtained that was maintained during the studied sparkling process. Chrome decreased from 20.7 (melon must) toward 0.8 to 1.2, which means a decline in the green color intensity, due to the filtration process and its clarification [32].

The TPC and antioxidant capacities (FRAP and TEAC) in enriched melon must were 173.6 mg GAE/L, 0.94 mmol Fe^+2^/L, and 0.47 mmol TE/L, respectively. Fermentation led to a decrease in those values, with 153 mg GAE/L being obtained in the still wine, and the antioxidant capacity was 0.73 mg Fe^+2^/L and 0.43 mmol TE/L. These values were similar for the sparkling melon wine during the eight weeks of storage, excepting the end of the storage time when an increase in TPC (182 mg GAE/L) and antioxidant activity (1.15 mg Fe^+2^/L and 0.78 mmol TE/L) was found, probably due to the interference of metabisulfite as an antioxidant agent [33], which was added in week 10.

### 3.2. Polar Compounds Quantification

#### 3.2.1. Individual Sugar and Organic Acids Content

The total sugar content for the melon must was 75.87 g/L; this high concentration was achieved after adding saccharose. In the first fermentation, the still melon wine obtained 4.82 g/L and the sparkling melon wine ranged from 4.51 to 9.10 g/L, with the lowest data being found after 10 weeks Table 4. This decrease in total sugars was the effect of the fermentation processes in which *S. cerevisiae*, which has the ability to ferment sugars, produced ethanol, CO_2_, and other secondary metabolites as the product of the alcoholic fermentation carried out [3]. The reduction in sugars, specifically glucose, in the sparkling melon wine after 10 weeks of storage shows that the fermentation process continued slowly inside the bottles [34]. Saccharose was only measured in the melon must and was completely consumed in the first alcoholic fermentation. A different trend was observed in the fructose and glucose concentrations, which decreased in the still wine with the first fermentation (2.95 and 1.87 g/L, respectively), but rose in the sparkling wine, due to the saccharose added (20 g/L) to initiate the second fermentation. After two weeks in the sparkling process, the fructose and glucose contents were 5.01 and 4.09 g/L, due to the *S. cerevisiae* Suc2 invertase enzyme which hydrolyzed extracellularly saccharose into glucose and fructose and increased the content of those monosaccharides [35]. After that, the fructose and glucose concentrations were remained stable throughout the sparkling process for 10 weeks Table 4. Similar trends were reported by Berbegal et al. [36] for sparkling wine with a dry yeast preparation.

Regarding total organic acids, the melon must presented a total of 8.40 g/L, decreasing slightly with the fermentations, 7.07 g/L in the still wine and 6.18 g/L in the sparkling wine (10 weeks). As mentioned in Section 2.1, the melon must was enriched with tartaric and malic acids (5 g/L, 1:1), providing a higher content for these organic acids. There were slight differences with a stabilization trend for tartaric acid throughout both fermentations and the sparkling melon wine storage (1.12–1.62 g/L). A similar trend in tartaric acid has been reported in other sparkling wines from grapes, in the 3rd and 12th months [37]. The malic acid concentration in the must (3.50 g/L) decreased after the first fermentation (2.37 g/L), due to malolactic fermentation, in which malic acid is transformed into lactic acid, normally by *Oenococcus*, *Lactobacillus* and *Pediococcus* species in typical grape wines, such production reduces the perceived acidity [4]. Malic acid continued to decrease slightly during the sparkling process (from 2.52 to 1.80 g/L). Succinic acid also decreased with the first fermentation, from 1.25 g/L in the must to 0.15 g/L in the still wine and remained stable during the sparkling process (0.16–0.28 g/L).

#### 3.2.2. Individual Amino Acids Content

Amino acids were quantified into four groups in accordance with Liang et al. [38]: bitter, sweet, umami, and other amino acids Table 5. The bitter amino acids included histidine, arginine, leucine, lysine, valine, phenylalanine, and isoleucine; sweet amino acids consisted of glycine, alanine, proline, serine, threonine, methionine, and cysteine; the umami amino acids were aspartic acid and glutamate; and other amino acids such as tyrosine, asparagine, glutamine, tryptophan, and cistin as the dimerization of two cysteines (Cys-Cys). The concentration of total amino acids in the melon must was 33.06 μM, although this dropped with the first alcoholic fermentation (18.31 μM) and was stable during the sparkling wine process (15.10 to 16.29 μM). This reduction was caused by the yeasts, whose population increased or retained a high viability during that period, and the use of amino acids for protein, RNA, and DNA synthesis, and storage as a reserve in vacuoles [34]. This trend was mainly detected in the total bitter amino acids, with arginine, phenylalanine, and histidine being the most abundant amino acids in that group. Arginine and phenylalanine decreased by 60 to 70% in this first fermentation and remained stable during the sparkling process. This behavior has been previously reported in wine during alcoholic fermentation by Wang et al. [39], who noted that these amino acids were consumed by the yeasts during the fermentation process as a nitrogen source. By contrast, histidine did not suffer a drop after fermentation and ranged between 5.54 and 6.45 μM during the experiment.

The total sweet amino acids rose from 0.92 μM in the must to 1.73 μM in the still wine, after the first fermentation, and slightly increased to 1.90 μM after two weeks of sparkling wine but decreased in the following weeks. The main sweet amino acid was alanine whose concentration changed following the same trend as mentioned for the total sweet amino acids. Some authors have suggested that the yeast’s own autolysis contributes to increase the amino acids content [39].

In the total umami amino acids, aspartate showed the highest concentration, rising from 0.07 μM to 1.01 μM in the first fermentation wine and decreasing to 0.84 μM at the end of the sparkling process. Other amino acids such as tyrosine, asparagine, and glutamine presented very low concentrations in the still wine and during the sparkling process (<0.08 μM). Tryptophan decreased from 0.88 mM in the must to 0.25 mM in the still wine and ranged from 0.14 to 0.22 mM during the sparkling process. By contrast, cistin (dimerization of cisteine) increased from the must to the still wine (0.10 to 0.26 mM, respectively) and decreased during the sparkling process (0.26 to 0.10 mM).

### 3.3. Volatile Compounds in Melon Wines and Sparkling Wine

**Figure 2:**
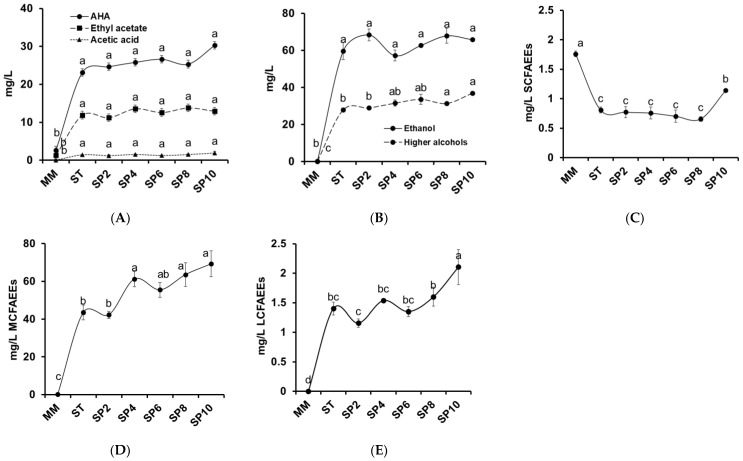
Evolution of aroma volatiles for enriched must, still wine, and during the sparkling process of melon-based wine (10 weeks). Must (MM); Still wine (ST); Sparkling wine (SP) in weeks (2, 4, 6, 8, and 10). (**A**) Acetate higher alcohol (AHA), ethyl acetate and acetic acid. (**B**) Ethanol and higher alcohols. (**C**) Short-chain fatty acid ethyl ester (SCFAEEs). (**D**) Medium-chain fatty acid ethyl ester (MCFAEEs). (**E**) Long-chain fatty acid ethyl ester (LCFAEEs). Different letters in the same image indicate significant differences (*p* < 0.05).

The volatile compounds identified in these melon wines were classified as: ethyl acetate, acetic acid, acetate higher alcohols (AHA), short-chain (C3-C5) fatty acid ethyl ester (SCFAEE), medium-chain (C6-C12) fatty acid ethyl ester (MCFAEE), long-chain (C13-C22) fatty acid ethyl ester (LCFAEE), other esters, ethanol, higher alcohols (HA), and other compounds. The aroma evolution during the first alcoholic fermentation and the sparkling process is shown in Figure 2, except for other ester and other compound groups which can be consulted in Table 6.

Acetate esters and acetic acid are presented in Figure 2A. Initially, the content of ethyl acetate in the must was low (1.30 mg/L) compared to its increment in the first alcoholic fermentation (11.88 mg/L) and stabilization during the sparkling process (11.22 to 13.79 mg/L). These acetate esters, common in wine, are responsible for the negative effect on flavor and spoilage character when they exceed 150 to 200 mg/L [40], far higher than our values. However, acetate esters under 80 mg/L concentration may improve the aroma of wine [41]. Acetic acid was not detected in must but appeared with the fermentations, 1.23 to 1.95 mg/L.

**Figure 3:**
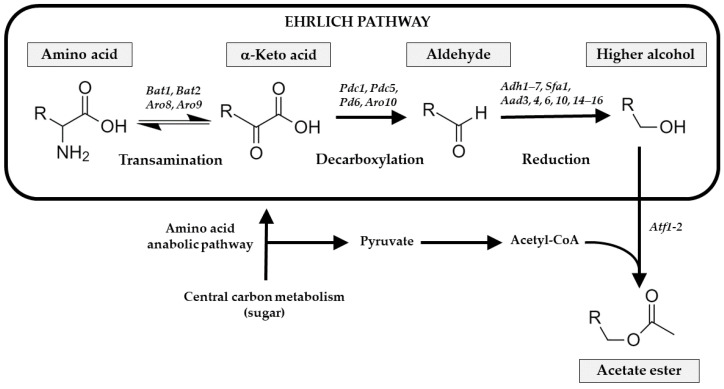
Ehrlich pathway (square) in Saccharomyces cerevisiae, biochemistry and main genes encoding enzymes (italics) involved in this catabolic pathway. It consists of three stages in which higher alcohols are produced from assimilated amino acids. In the first step, amino acids are deaminated in a reversible transamination reaction to the corresponding α-keto acids, followed by a decarboxylation in the second step of α-keto acids to aldehydes. In the third step, the reduction of aldehydes to the corresponding alcohol by alcohol dehydrogenases catalyzes this last step of the Ehrlich pathway. The higher alcohols can be esterified to produce the equivalent acetate esters once they have been synthesized. The biosynthesis of amino acids from a carbon source, or the anabolic pathway, may also provide the α-keto acid.

AHA increased from 2.57 mg/L in the must to 23.14 in the still wine and to 27.81 mg/L in the sparkling wine (10 weeks). Isoamyl acetate was the principal AHA Table 6, representing around 60% of all AHA compound. In wines, this compound is responsible for providing a fruity character in the wine aroma [42]. Zhang et al. [43] found that *S. cerevisiae* catabolites the leucine into 3-methyl-butyraldehyde and isoamyl alcohol, via the Ehrlich pathway Figure 3, and the final esterification step with active acetic acid or acetyl-CoA to isoamyl acetate; its yields are regulated by the initial concentration of leucine. Our results follow that trend, with an increased concentration of isoamyl acetate, showing a negative correlation with the leucine concentration. 

The next principal AHA compounds were 2-phenylethyl acetate and isobutyl acetate, which increased during the first fermentation (3.84 mg/L) and the sparkling process (5.13 mg/L). As with the isoamyl acetate and leucine correlation, a similar scenario occurred with 2-phenylethyl acetate and phenylalanine, which *S. cerevisiae* catabolized into 2-phenylethanol and 2-phenylethyl acetate [30], and this metabolic trend is shown in the decrement of phenylalanine and the increment in its alcoholic and acetate metabolites. Another AHA that increased during the first alcoholic fermentation was 3,6-nonadien-1-yl acetate, probably derived from 3,6-nonadien-1-ol due to the activity of alcohol acetyltransferases, which catalyzed alcohols and acetyl-coenzyme A for the formation of acetate ester [44].

Ethanol and higher alcohols are presented in Figure 2B. The melon wine fermentations converted sugar into alcohol; an ethanol concentration of 59.73 mg/L was obtained in the still wine, and it reached 65.83 mg/L in the sparkling wine (10 weeks). 

Higher alcohols also increased with a slight rise during the sparkling process, principally isoamyl alcohol, derived from the deamination, decarboxylation, and reduction of leucine as explained above, via the Ehrlich pathway. Isoamyl alcohol was not detected in the must Table 6 and developed with the first fermentation and remained stable throughout the sparkling processes (18.70 to 24.17 mg/L). 2-Phenylethanol was the third most abundant higher alcohol, derived from phenylalanine, and ranged from 4.71 mg/L in the still wine to 8.16 mg/L in the sparkling processes (10 weeks). Isobutyl alcohol and 2,3-butanediol were identified with alcoholic fermentation. As reported by Wess et al. [45], *S. cerevisiae* metabolizes valine amino acid by the Ehrlich pathway to produce isobutyl alcohol in the cytosol. Another alcohol that competes with the ethanol pathway is 2,3-butanediol which, from 2-acetolactate after a spontaneous decarboxylation and followed by oxidation by butanediol dehydrogenases, is reduced to 2,3-butanediol [45]; this was detected in very low concentrations in both fermentation processes. Another higher alcohol identified was (E,Z)-3,6-nonadien-1-ol, which was found in the must, as reported in other melon cultivars [46] and doubled its concentration with fermentations. 

An aromatic higher alcohol, 2,4-di-tert-butylphenol increased from 1.75 to 2.35 mg/L after the first alcoholic fermentation, probably due to the oxidation of 1,3-di-tertbutyl-benzene, which was also identified Table 6.

Fatty acid ethyl esters Figure 2C–E were produced by *S. cerevisiae* during the first alcoholic fermentation and sparkling process and determined the complex flavor and fruity aroma, obtained from the condensation of acyl-CoA with ethanol, catalyzed by ethanol-O-acyl transferase [47].

The most abundant group of ethyl esters was MCFAEEs, followed by LCFAEEs. Both groups increased during the first fermentation and sparkling process, ranging from 43.25 to 69.23 mg/L and 1.16 to 2.11 mg/L, respectively. In contrast, SCFAEEs decreased with the first fermentation (0.81 mg/L) and showed a slight increase at the end of the studied sparkling process (1.13 mg/L). Five SCFAEEs were identified in the must Table 6, principally ethyl 2-methyl butyrate and ethyl butanoate, followed by ethyl isobutyrate, ethyl propanoate, and ethyl pentanoate. These last three compounds decreased to a non-detected limit after the first alcoholic fermentation. However, ethyl 2-methyl butyrate decreased from 0.77 mg/L in the must to 0.17–0.32 mg/L after the first fermentation and sparkling process. Ethyl butanoate ranged from 0.46 to 0.68 mg/L during the experiment, increasing to 0.82 mg/L after 10 weeks. Generally, ethyl 2-methyl butyrate and ethyl butanoate are commonly identified in a large range of white and rosé wines fermented with *S. cerevisiae* [48].

Regarding MCFAEEs, five ethyl esters were identified throughout the experiment, with a gradual increase during the first alcoholic fermentation and sparkling process, with the maximum concentration being obtained at the end of the experiment (69.23 mg/L) Figure 2D. Ethyl hexanoate was the only compound identified in the must. Instead, the first alcoholic fermentation provided the formation of new aromas such as ethyl octanoate, ethyl decanoate, ethyl hexanoate, ethyl 9-decenoate and ethyl laurate, with octanoate and decanoate being the compounds found in the highest concentrations. All these MCFAEE volatile compounds have been identified in other alcoholic fermentative beverages such as wines from grapes [48,49] and kiwi wines [6].

Concerning LCFAEEs, ethyl tetradecanoate and ethyl hexadecanoate were not identified in the must but developed with alcoholic fermentation. The concentration of these compounds was low, with a stable trend throughout the sparkling process (0.57 to 0.75 mg/L, and 0.59–1.39 mg/L, respectively) Table 6. Both compounds have been reported in other sparkling grape wines [50] and fruit-based wines such as pomegranate and kiwi [6,51].

### 3.4. Odor Activity Value (OAV) and Relative Odor Contribution (ROC)

**Figure 4:**
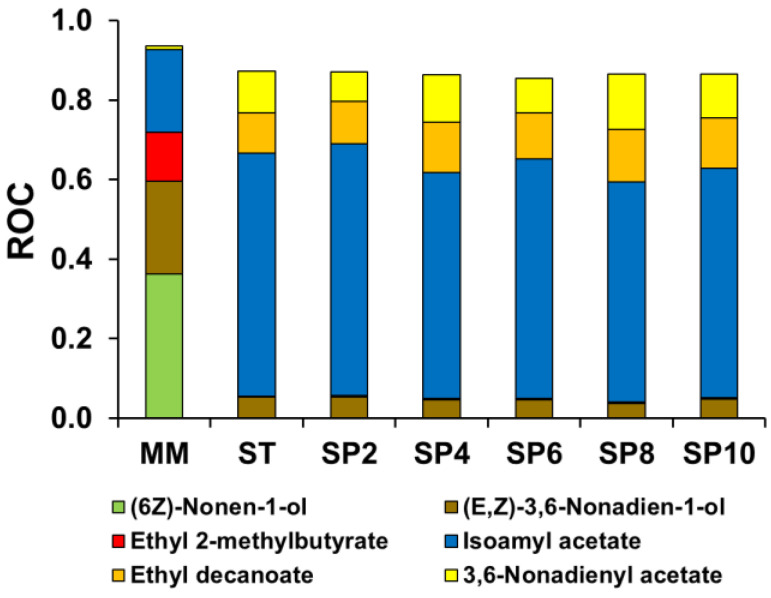
Evolution of principal relative odor contribution value (ROC > 0.1) in enriched must, still wine, and during the sparkling process of melon-based wine (10 weeks). MM: Must. ST: Still wine. SP: Sparkling wine in weeks (2, 4, 6, 8, and 10 weeks).

Table 7 shows the 15 individual volatile compounds which had at least OAV > 1 and contributed to the aroma of the melon-based wine. Figure 4 illustrates the principal aromas with a ROC value above 0.1. In the AHA groups, only four compounds exceeded the odor threshold: isobutyl acetate, isoamyl acetate, 3,6-nonadienyl acetate, and 2-phenylethyl acetate. As mentioned before, these compounds increased with the first alcoholic fermentation and sparkling process. Isoamyl acetate, followed by 3,6-nonadienyl acetate, were the AHA compounds with the highest OAV values in the must, with an increase in the alcoholic fermentations providing sweet banana and fruity odors in the still and sparkling wines Table 7. Isoamyl acetate, with an initial (must) OAV value of 17.7, increased to 481 in the still wine and 647 at the end of the sparkling process and its ROC increased from 0.2 (must) to 0.55–0.61, during the first alcoholic fermentation and the sparkling process, meaning that the aroma was a major contribution to the melon-based wine.

Regarding the SCFAEEs, two volatile compounds contributed to the aroma of the melon-based wines: ethyl butanoate and ethyl 2-methyl butyrate. The first aroma was detected in the must (1.1), the still wine (1.1) and at the end of the sparkling process (1.4). Ethyl 2-methyl butyrate decreased its OAV value from 10.5 in the must to 2.3 in the still wine and increased to 4.3, at the end of the sparkling period studied. Both aromas provide a sweet and fruity connotation; however, only ethyl 2-methylbutyrate reached a ROC > 0.1 value in the must, after which other aromas became more relevant Figure 4.

For the MCFAEEs, the trend of these fatty acid ethyl esters was, after AHA, the group with the largest increment in OAV; it increased with the fermentation process, and its evolution followed a similar pattern to other grape and fruit wines [5,52]. Ethyl hexanoate was the only MCFAEEs, with OAV > 1, present in the must (1.9), which increased with the fermentations and remained stable, ranging from 20.7 to 24.1. However, the compounds with high OAV were ethyl decanoate (78.9 to 141.9), whose odor description corresponds to sweet, fruity, nuts, and dried fruit, and ethyl octanoate (34.1 to 74.3) which provides pineapple, pear, and a soapy odor. The highest levels were obtained at the end of the sparkling process. However, only ethyl decanoate managed to contribute a ROC > 0.1, between 0.10 to 0.13, in the still and sparkling wines. Ethyl 9-decenoate had a value between 11.2 to 18.9 after alcoholic fermentations, providing a floral odor with a ROC > 0.1 Figure 4. For LCFAEEs group, only ethyl tetradecanoate had an OAV > 1 after first fermentation (1.5) and ranged 1.10—1.50 during the sparkling process. Regarding ethyl hexadecanoate, it only reached an OAV > 1 with 1.39 at 10 weeks of the sparkling process. Both volatile aromas contributed a fruity and fatty odor but with low contribution to the aroma of the still and sparkling melon wine (ROC > 0.1).

(6Z)-Nonen-1-ol and (E,Z)-3,6-nonadien-1-ol are common aromas in some melon cultivars [46], and they were the only HA that reached their odor threshold with a melon-like and sweet odor description in the initial must (32.2 and 20.2, respectively), and with the highest ROC values of 0.4 and 0.23, respectively. After the first alcoholic fermentation, the contribution of (6Z)-nonen-1-ol disappeared but that of (E,Z)-3,6-nonadien-1-ol increased, with an OAV value of 33.6–56.44 during the experiment, with an ROC value of around 0.05.

### 3.5. Sensory Evaluation of Final Sparkling Wine

The sensory evaluation of the sparkling melon, after 10 weeks in the bottle, is shown in Table 8. In accordance with the categories described by OIV (2009), this sparkling melon-based wine, obtained a total mark of 92.11 points, after the sum of all markers (visual, nose, taste, and harmony), achieving the “Grand Gold” category (>92 points). Considering visual aspects (14.44), the judges rated the sparkling wine as limpid to excellent limpidity, which means a good visual impression without cloudiness. Regarding the sensation perceived in the nose, melon-based wine had a very total absence of defects in genuineness (5.78), very strong qualitative intensity (7.78), and a very-to-excellent impression of quality (15.11). These results refer to the intensity of OAV Table 7, such as isoamyl acetate, ethyl decanoate, 3,6-nonadienyl acetate and (E,Z)-3,6-nonadien-1-ol, which are the most relevant volatile aromas providing the highest ROC values that contributed to the complexity and fruity-aroma character in this sparkling melon wine. Concerning taste values (39.11), sparkling melon wine was characterized as having a total absence of defects in genuineness, strong intensity (7.11), a very good persistence of residual olfactory-gustatory sensation of flavors (7.22), and a very good impression for the taste quality (19.33). These results suggest that this melon wine is absent in oxidation flavor, volatile acidity, and a negative flavor that affects the taste and mouthfeel. Finally, the judges marked a very good general impression (9.89), which along with the total marks received, defined our sparkling melon-based wine as a potential marketable and novel product.

## 4. Conclusions

In this study, a novel melon-based sparkling wine, using melons that failed to meet cosmetic standards, was developed and characterized. In this sparkling melon wine, a second fermentation takes place in the bottle, from a still wine (first fermentation). In our case, during the second fermentation, *S. cerevisiae* provided significant physico-chemical and aroma changes. Certain amino acids contributed to the transformation and increase in some volatile compounds via the Ehrlich pathway, in this case, leucine to isoamyl alcohol, valine to isobutyl alcohol and phenylalanine to phenethyl alcohol. Furthermore, during the second fermentation principally medium- and long-chain fatty acid ethyl ester increased and reached its odor threshold and contributed to the sweet, fruity, banana, tropical, nutty and melon aroma character, mainly by isoamyl acetate, ethyl decanoate, 3,6-nonadienyl acetate, and (E,Z)-3,6-nonadien-1-ol, with the highest ROC values during the sparkling process. In summary, this study is an example of how the agriculture sector should support a circular economy model with by-products revalorization such as fresh fruit that does not reach aesthetic standards, providing a novel fruit-based wine with a characteristic and distinctive aroma, good sensory acceptance and with market potential.

## Figures and Tables

**Table 1:** Amino acids and molecular formula, molecular mass (MS), and qualifier fragment (MS/MS).

Amino Acid	Abbreviature	Formula	MS	MS/MS
Glycine	Gly	C_2_H_5_NO_2_	76.0393	
Alanine	Ala	C_3_H_7_NO_2_	90.055	
Serine	Ser	C_3_H_7_NO_3_	106.0499	88.0389
Proline	Pro	C_5_H_9_NO_2_	116.0706	70.0645
Valine	Val	C_5_H_11_NO_2_	118.0863	72.0801
Threonine	Thr	C_4_H_9_NO_3_	120.0655	74.0601
Cysteine	Cys	C_3_H_7_NO_2_S	122.027	76.0208
Isoleucine	Ile	C_6_H_13_NO_2_	132.1019	86.0959
Leucine	Leu	C_6_H_13_NO_2_	132.1019	86.0959
Asparagine	Asn	C_4_H_8_N_2_O_3_	133.0608	87.0551
Aspartate	Asp	C_4_H_7_NO_4_	134.0448	88.0387
Glutamine	Gln	C_5_H_10_N_2_O_3_	147.0764	84.0442
Lysine	Lys	C_6_H_14_N_2_O_2_	147.1128	84.0802
Glutamate	Glu	C_5_H_9_NO_4_	148.0604	84.0442
Methionine	Met	C_5_H_11_NO_2_S	150.0583	104.0527
Histidine	His	C_6_H_9_N_3_O_2_	156.0768	110.0711
Phenylalanine	Phe	C_9_H_11_NO_2_	166.0863	120.0807
Arginine	Arg	C_6_H_14_N_4_O_2_	175.119	116.0703
Tyrosine	Tyr	C_9_H_11_NO_3_	182.0812	165.0540
Tryptophane	Trp	C_11_H_12_N_2_O_2_	205.0972	188.0699
Cystine	Cys-Cys	C_6_H_12_N_2_O_4_S_2_	241.0311	122.0273

**Table 2:** Organoleptical characteristics and definitions used in sensory evaluation.

Organoleptical Characteristic	Definition	Range (Excellent to Inadequate)
**Visual**	**Discrimination of differences in outside world with sensory impressions from visible light rays**	**(15–3)**
Limpidity	Measure of cloudiness.	(5–1)
Aspect other than limpidity	Determine the full spectrum of visible properties of a product	(10–2)
**Nose**	**Sensations perceived by the olfactory organ when stimulated by certain volatile substances**	**(30–12)**
Genuineness	Measure degree of sensation perceived (magnitude) by the nose, of a viticulture, oenological defect of product	(6–2)
Positive intensity	Degree (magnitude) of full spectrum of qualitative odors perceived by nose.	(8–2)
Quality	Spectrum of properties and characteristics of a wine that gives an aptitude to satisfy nose, implicit or expressed needs	(16–8)
**Taste**	**Full spectrum of sensations perceived with wine mouthfeel.**	**(44–18)**
Genuineness	Measure degree of sensation perceived (magnitude) by the taste, of a viticulture, oenological defect of product	(6–2)
Positive intensity	Degree (magnitude) of full spectrum of qualitative odors perceived by taste.	(8–2)
Harmonious persistence	To measure the length of residual olfactory-gustatory sensation, corresponding to the sensation perceived when the product is in mouth and length of time is measured.	(8–4)
Quality	Degree (magnitude) of full spectrum of qualitative odors perceived by taste	(22–10)
**Harmony-Overall judgement**	**Corresponds to overall appraisal of a product.**	**(11–7)**

**Table 3:** Physico-chemical parameters of enriched must, still wine, and during the sparkling process of melon-based wine (10 weeks).

Parameter	Must	Still Wine	Sparkling Wine (Weeks)				
			2	4	6	8	10
Alcohol (%; *v*/*v*)		12.3 ± 0.1 ^ns^	12.3 ± 0.0	12.5 ± 0.1	12.4 ± 0.0	12.3 ± 0.1	12.4 ± 0.1
TSS (°Brix)	21.3 ^z^ ± 0.7 ^a^	8.7 ± 0.0 ^b^	8.6 ± 0.0 ^b^	8.5 ± 0.2 ^b^	8.5 ± 0.0 ^b^	8.5 ± 0.0 ^b^	8.5 ± 0.0 ^b^
Residual sugar (g/L)	90.55 ± 3.49 ^a^	5.72 ± 0.73 ^b^	10.23 ± 1.25 ^b^	6.03 ± 2.12 ^b^	8.09 ± 0.04 ^b^	7.20 ± 0.52 ^b^	5.38 ± 0.02 ^b^
Sugar-free extract (g/L)	142.0 ± 4.4 ^a^	25.1 ± 0.9 ^b^	20.6 ± 1.2 ^b^	23.2 ± 1.5 ^b^	22.8 ± 0.1 ^b^	22.4 ± 0.7 ^b^	24.6 ± 0.0 ^b^
pH	4.4 ± 0.1 ^a^	3.9 ± 0.0 ^b^	4.0 ± 0.0 ^b^	4.0 ± 0.0 ^b^	4.0 ± 0.0 ^b^	4.1 ± 0.0 ^b^	4.1 ± 0.0 ^b^
TA (g/L)	6.44 ± 0.24 ^ns^	5.62 ± 0.13	6.40 ± 0.06	6.63 ± 0.08	6.53 ± 0.01	6.51 ± 0.48	6.76 ± 0.17
Volatile acidity (g/L)	0.10 ± 0.01 ^c^	0.27 ± 0.02 ^ab^	0.28 ± 0.00 ^ab^	0.35 ± 0.02 ^a^	0.32 ± 0.00 ^ab^	0.23 ± 0.01 ^b^	0.34 ± 0.05 ^a^
SO_2_ free (mg/L)	11.5 ± 0.0 ^b^	0.0 ± 0.0 ^c^	0.0 ± 0.0 ^c^	0.0 ± 0.0 ^c^	0.0 ± 0.0 ^c^	0.0 ± 0.00 ^c^	12.8 ± 0.7 ^a^
SO_2_ total (mg/L)	25.6 ± 0.0 ^b^	26.2 ± 0.9 ^b^	26.9 ± 0.4 ^b^	25.0 ± 2.1 ^b^	25.1 ± 0.2 ^b^	22.7 ± 3.4 ^b^	75.7 ± 2.3 ^a^
L*	39.2 ± 0.2 ^a^	33.5 ± 0.2 ^bc^	33.8 ± 0.0 ^bc^	33.4 ± 0 ^c^	33.6 ± 0.1 ^bc^	33.6 ± 0.0 ^bc^	33.9 ± 0.1 ^b^
°Hue	109.0 ± 0,5 ^b^	178.5 ± 0.0 ^a^	178.5 ± 0.0 ^a^	178.6 ± 0.1 ^a^	178.5 ± 0.0 ^a^	178.5 ± 0.0 ^a^	178.6 ± 0.0 ^a^
Chroma	20.7 ± 0.6 ^a^	0.9 ± 0.0 ^b^	0.8 ± 0.0 ^b^	1.0 ± 0.1 ^b^	0.8 ± 0.1 ^b^	1.2 ± 0.1 ^b^	1.1 ± 0.1 ^b^
TPC	173.6 ± 4.6 ^ab^	153.9 ± 2.4 ^c^	164.6 ± 3.4 ^bc^	157.6 ± 2.0 ^c^	158.4 ± 0.1 ^c^	156.2 ± 1.3 ^c^	182.4 ± 3.0 ^a^
FRAP	0.94 ± 0.01 ^b^	0.73 ± 0.02 ^c^	0.76 ± 0.01 ^c^	0.72 ± 0.03 ^c^	0.66 ± 0.01 ^c^	0.63 ± 0.02 ^c^	1.15 ± 0.08 ^a^
TEAC	0.47 ± 0.01 ^b^	0.42 ± 0.02 ^b^	0.46 ± 0.01 ^b^	0.45 ± 0.01 ^b^	0.42 ± 0.00 ^b^	0.43 ± 0.01 ^b^	0.78 ± 0.05 ^a^

^z^ Means (*n* = 3 ± SE). TSS: Total soluble solids. TA (g TE/L): g tartaric acid equivalent per liter. Volatile acidity (g/L): g acetic acid equivalent per liter. TPC: Total phenolic compounds, mg gallic acid equivalent/L (mg GAE/L). FRAP: Ferric reducing antioxidant capacity, mmol Fe^+2^ equivalent/L (mmol Fe^+2^/L). TEAC: Trolox equivalent antioxidant capacity, mmol Trolox equivalent/L (mmol TE/L). Different letters in the same row indicate significant differences (*p* < 0.01) among must, still wine, and sparkling process. ns: statistically non-significant differences.

**Table 4:** Evolution of individual sugars and organic acids of enriched must, still wine, and during the sparkling process of melon-based wine (10 weeks).

Polar Compounds	Must	Still Wine	Sparkling Wine (Weeks)				
			2	4	6	8	10
***Individual sugar (g/L)***							
Saccharose	53.63 ^Z^ ± 2.58 ^a^	n.d.	n.d.	n.d.	n.d.	n.d.	n.d.
Fructose	10.90 ± 0.55 ^a^	2.95 ± 0.40 ^c^	5.01 ± 0.73 ^b^	3.27 ± 0.03 ^c^	4.29 ± 0.02 ^bc^	4.05 ± 0.66 ^bc^	3.10 ± 0.02 ^c^
Glucose	11.34 ± 0.64 ^a^	1.87 ± 0.36 ^c^	4.09 ± 0.83 ^b^	1.74 ± 0.01 ^c^	2.70 ± 0.02 ^bc^	2.27 ± 0.19 ^c^	1.41 ± 0.03 ^c^
**∑ Total**	**75.87 ± 3.77 ^a^**	**4.82 ± 0.75 ^b^**	**9.10 ± 1.55 ^b^**	**5.01 ± 0.04 ^b^**	**6.98 ± 0.04 ^b^**	**6.32 ± 0.59 ^b^**	**4.51 ± 0.04 ^b^**
***Organic acids (g/L)***							
Fumaric acid (mg/L)	11.49 ± 1.59 ^a^	7.66 ± 1.46 ^bc^	10.67 ± 1.53 ^ab^	9.80 ± 0.63 ^abc^	11.72 ± 0.05 ^a^	6.84 ± 0.13 ^b^	9.66 ± 1.45 ^abc^
Succinic acid	1.25 ± 0.20 ^a^	0.15 ± 0.02 ^c^	0.28 ± 0.03 ^b^	0.20 ± 0.00 ^c^	0.25 ± 0.00 ^bc^	0.16 ± 0.02 ^c^	0.19 ± 0.01 ^c^
Malic acid	3.50 ± 0.43 ^a^	2.37 ± 0.03 ^b^	2.52 ± 0.27 ^b^	2.39 ± 0.09 ^b^	1.80 ± 0.30 ^b^	1.88 ± 0.09 ^b^	1.80 ± 0.08 ^b^
Citric acid	2.07 ± 0.24 ^b^	2.92 ± 0.22 ^ab^	3.19 ± 0.50 ^a^	2.83 ± 0.04 ^ab^	3.12 ± 0.11 ^a^	2.93 ± 0.40 ^ab^	2.59 ± 0.39 ^ab^
Tartaric acid	1.56 ± 0.22 ^b^	1.62 ± 0.37 ^ab^	1.31 ± 0.23 ^b^	1.57 ± 0.07 ^b^	1.12 ± 0.05 ^b^	1.97 ± 0.25 ^a^	1.59 ± 0.05 ^b^
**∑ Total**	**8.40 ± 1.08 ^ns^**	**7.07 ± 0.32**	**7.31 ± 0.02**	**7.00 ± 0.15**	**6.31 ± 0.46**	**6.95 ± 0.51**	**6.18 ± 0.43**

Mean ± SE (*n* = 3). Different letters in the same row indicate significant differences (*p* < 0.05) among must, still wine, and sparkling process. n.d.: not detected. ns: statistically non-significant differences.

**Table 5:** Evolution of amino acids (mM) of enriched must, still wine, and during the sparkling process of melon-based wine (10 weeks).

Polar Compounds		Must	Still Wine	Sparkling Wine (Weeks)				
				2	4	6	8	10
**TBAA**	Histidine	6.19 ^z^ ± 0.14 ^ab^	6.45 ± 0.21 ^a^	5.54 ± 0.04 ^c^	5.97 ± 0.18 ^bc^	6.02 ± 0.10 ^ab^	6.19 ± 0.19 ^ab^	6.22 ± 0.22 ^ab^
	Arginine	13.67 ± 0.51 ^a^	3.97 ± 0.08 ^b^	3.61 ± 0.00 ^b^	3.79 ± 0.08 ^b^	3.91 ± 0.02 ^b^	3.86 ± 0.05 ^b^	3.94 ± 0.01 ^b^
	Leucine	0.26 ± 0.08 ^a^	0.05 ± 0.01 ^b^	0.05 ± 0.00 ^b^	0.04 ± 0.00 ^b^	0.04 ± 0.00 ^b^	0.03 ± 0.00 ^b^	0.03 ± 0.00 ^b^
	Lysine	0.13 ± 0.01 ^a^	0.12 ± 0.00 ^a^	0.09 ± 0.00 ^b^	0.12 ± 0.01 ^a^	0.06 ± 0.01 ^c^	0.08 ± 0.01 ^bc^	0.06 ± 0.01 ^c^
	Valine	0.04 ± 0.00 ^ns^	0.05 ± 0.00	0.05 ± 0.00	0.04 ± 0.01	0.04 ± 0.00	0.04 ± 0.00	0.04 ± 0.00
	Phenylalanine	10.44 ± 1.45 ^a^	3.95 ± 0.05 ^b^	3.27 ± 0.03 ^bc^	2.84 ± 0.15 ^bc^	2.50 ± 0.05 ^bc^	2.15 ± 0.05 ^c^	2.12 ± 0.01 ^c^
	Isoleucine	0.13 ± 0.05 ^a^	0.01 ± 0.00 ^b^	0.02 ± 0.00 ^b^	0.02 ± 0.00 ^b^	0.02 ± 0.00 ^b^	0.02 ± 0.00 ^b^	0.02 ± 0.00 ^b^
	**∑ TBAA**	**30.87 ± 2.0 ^a^**	**14.60 ± 0.33 ^b^**	**12.63 ± 0.08 ^b^**	**12.83 ± 0.28 ^b^**	**12.59 ± 0.14 ^b^**	**12.38 ± 0.10 ^b^**	**12.45 ± 0.21 ^b^**
**TSAA**	Glycine	0.07 ± 0.01 ^c^	0.32 ± 0.03 ^a^	0.26 ± 0.00 ^b^	0.28 ± 0.01 ^ab^	0.29 ± 0.03 ^ab^	0.27 ± 0.01 ^ab^	0.09 ± 0.01 ^c^
	Alanine	0.67 ± 0.07 ^ns^	0.80 ± 0.06	0.86 ± 0.01	0.73 ± 0.03	0.73 ± 0.02	0.74 ± 0.03	0.67 ± 0.03
	Proline	0.07 ± 0.01 ^ns^	0.16 ± 0.09	0.22 ± 0.09	0.05 ± 0.06	0.05 ± 0.00	0.04 ± 0.00	0.05 ± 0.00
	Serine	0.01 ± 0.00 ^d^	0.27 ± 0.01 ^a^	0.25 ± 0.00 ^b^	0.22 ± 0.01 ^c^	0.22 ± 0.00 ^c^	0.21 ± 0.00 ^c^	0.21 ± 0.00 ^c^
	Threonine	0.03 ± 0.00 ^bc^	0.03 ± 0.00 ^ab^	0.04 ± 0.00 ^a^	0.03 ± 0.00 ^bc^	0.03 ± 0.00 ^bc^	0.03 ± 0.00 ^bc^	0.03 ± 0.00 ^c^
	Metionine	0.14 ± 0.04 ^ns^	0.12 ± 0.00	0.13 ± 0.00	0.10 ± 0.01	0.09 ± 0.01	0.10 ± 0.01	0.08 ± 0.01
	Cysteine	0.01 ± 0.01 ^c^	0.34 ± 0.03 ^b^	0.41 ± 0.01 ^a^	0.31 ± 0.03 ^b^	0.32 ± 0.01 b	0.32 ± 0.00 ^b^	0.32 ± 0.03 ^b^
	**∑ TSAA**	**0.92 ± 0.11 ^c^**	**1.73 ± 0.12 ^a^**	**1.90 ± 0.09 ^a^**	**1.44 ± 0.13 ^b^**	**1.43 ± 0.02 ^b^**	**1.44 ± 0.04 ^b^**	**1.37 ± 0.03 ^b^**
**TUAA**	Aspartate	0.07 ± 0.06 ^c^	1.01 ± 0.04 ^a^	0.87 ± 0.01 ^abc^	0.87 ± 0.05 ^bc^	0.82 ± 0.04 ^b^	0.99 ± 0.05 ^ab^	0.84 ± 0.04 ^bc^
	Glutamate	0.00 ± 0.00 ^d^	0.02 ± 0.00 ^a^	0.01 ± 0.00 ^ab^	0.00 ± 0.00 ^d^	0.01 ± 0.00 ^b^	0.01 ± 0.00 ^bc^	0.01 ± 0.00 ^c^
	**∑ TUAA**	**0.07 ± 0.06 ^d^**	**1.03 ± 0.04 ^a^**	**0.89 ± 0.01 ^bc^**	**0.87 ± 0.05 ^c^**	**0.83 ± 0.04 ^c^**	**1.01 ± 0.06 ^ab^**	**0.85 ± 0.05 ^c^**
**Others**	Tyrosine	0.01 ± 0.00 ^b^	0.02 ± 0.00 ^a^	0.01 ± 0.00 ^a^	0.02 ± 0.00 ^a^	0.01 ± 0.00 ^a^	0.01 ± 0.00 ^a^	0.02 ± 0.00 ^a^
	Asparagine	0.00 ± 0.00 ^c^	0.08 ± 0.01 ^a^	0.08 ± 0.00 ^ab^	0.07 ± 0.01 ^ab^	0.07 ± 0.01 ^b^	0.08 ± 0.00 ^ab^	0.08 ± 0.00 ^ab^
	Glutamine	0.15 ± 0.02 ^a^	0.02 ± 0.00 ^b^	0.02 ± 0.01 ^b^	0.01 ± 0.00 ^b^	0.01 ± 0.00 ^b^	0.01 ± 0.00 ^b^	0.01 ± 0.00 ^b^
	Tryptophan	0.88 ± 0.08 ^a^	0.25 ± 0.01 ^b^	0.22 ± 0.00 ^bc^	0.20 ± 0.02 ^bc^	0.16 ± 0.01 ^bc^	0.16 ± 0.01 ^bc^	0.14 ± 0.00 ^c^
	Cistin	0.10 ± 0.00 ^d^	0.26 ± 0.02 ^a^	0.27 ± 0.01 ^a^	0.20 ± 0.02 ^b^	0.19 ± 0.00 ^b^	0.15 ± 0.01 ^c^	0.10 ± 0.01 ^d^
	**∑ Total AA**	**33.06 ± 2.18 ^a^**	**18.31 ± 0.49 ^b^**	**16.29 ± 0.03 ^bc^**	**15.92 ± 0.23 ^bc^**	**15.59 ± 0.09 ^c^**	**15.50 ± 0.17 ^c^**	**15.10 ± 0.15 ^c^**

Mean ± SE (*n* = 3). TBAA: Total bitter amino acids. TSAA: Total sweet amino acids. TUAA: Total umami amino acids. TAA: Total amino acids. Different letters in the same row indicate significant differences (*p* < 0.05) among must, still wine, and sparkling process. ns: statistically non-significant differences.

**Table 6:** Evolution of aroma volatiles (mg/L) in enriched must, still wine, and during the sparkling process of melon-based wine (10 weeks).

Group	RT	Compounds	KI	Must	Still Wine	Sparkling Wine (Weeks)				
						2	4	6	8	10
	2.465	Ethyl acetate	<1100	1.30 ± 0.13 ^b^	11.88 ± 1.88 ^a^	11.22 ± 1.01 ^a^	13.57 ± 1.96 ^a^	12.60 ± 1.62 ^a^	13.79 ± 2.51 ^a^	12.92 ± 0.93 ^a^
	21.181	Acetic acid	1436	n.d.	1.46 ± 0.04 ^ab^	1.23 ± 0.24 ^b^	1.56 ± 0.08 ^ab^	1.27 ± 0.06 ^b^	1.53 ± 0.09 ^ab^	1.95 ± 0.40 ^a^
**AHA**	**3.785**	Isobutyl acetate	<1100	1.11 ± 0.11 ^c^	3.50 ± 0.17 ^b^	4.76 ± 0.71 ^ab^	4.71 ± 0.64 ^ab^	6.75 ± 1.44 ^a^	4.19 ± 0.29 ^b^	3.91 ± 0.41 ^b^
	4.816	Butyl acetate	<1100	0.24 ± 0.02 ^a^	0.12 ± 0.01 ^b^	n.d.	n.d.	n.d.	n.d.	n.d.
	5.910	Isoamyl acetate	<1100	0.53 ± 0.03 ^c^	14.43 ± 0.36 ^b^	14.88 ± 0.06 ^b^	15.39 ± 1.03 ^ab^	14.51 ± 1.19 ^b^	15.10 ± 0.96 ^b^	19.40 ± 2.80 ^a^
	7.439	Pentyl acetate	<1100	0.02 ± 0.00	n.d.	n.d.	n.d.	n.d.	n.d.	n.d.
	11.531	Hexyl acetate	1237	0.25 ± 0.03	n.d.	n.d.	n.d.	n.d.	n.d.	n.d.
	13.563	3-Hexenyl acetate	1286	0.10 ± 0.01	n.d.	n.d.	n.d.	n.d.	n.d.	n.d.
	23.062	2,3-Butanediol, diacetate	1470	0.07 ± 0.01	n.d.	n.d.	n.d.	n.d.	n.d.	n.d.
	25.216	2,3-Butanediol, diacetate	1506	0.05 ± 0.00	n.d.	n.d.	n.d.	n.d.	n.d.	n.d.
	32.218	3,6-Nonadienyl-acetate	1598	0.01 ± 0.00 ^c^	1.25 ± 0.01 ^ab^	0.89 ± 0.39 ^bc^	1.62 ± 0.26 ^ab^	1.09 ± 0.34 ^b^	1.98 ± 0.46 ^a^	1.83 ± 0.27 ^ab^
	33.439	Phenylmethyl acetate	1612	0.16 ± 0.02	n.d.	n.d.	n.d.	n.d.	n.d.	n.d.
	35.311	2-Phenylethyl acetate	1843	0.03 ± 0.00 ^c^	3.84 ± 0.33 ^b^	4.10 ± 0.24 ^ab^	4.07 ± 0.31 ^ab^	4.24 ± 0.45 ^ab^	4.04 ± 0.16 ^b^	5.13 ± 0.60 ^a^
		**∑ AHA**		2.57 ± 0.19 ^c^	23.14 ± 0.59 ^b^	24.63 ± 0.14 ^ab^	25.79 ± 1.04 ^ab^	23.30 ± 0.45 ^b^	25.31 ± 1.54 ^ab^	27.81 ± 2.83 ^a^
**SCFAEE**	**3.063**	Ethyl propanoate	<1100	0.08 ± 0.01	n.d.	n.d.	n.d.	n.d.	n.d.	n.d.
	3.160	Ethyl isobutyrate	<1100	0.39 ± 0.04	n.d.	n.d.	n.d.	n.d.	n.d.	n.d.
	4.160	Ethyl butanoate	<1100	0.68 ± 0.07 ^a^	0.64 ± 0.04 ^b^	0.57 ± 0.12 ^b^	0.55 ± 0.08 ^b^	0.51 ± 0.09 ^b^	0.46 ± 0.03 ^b^	0.82 ± 0.09 ^b^
	4.451	Ethyl 2-methylbutyrate	<1100	0.77 ± 0.07 ^a^	0.17 ± 0.01 ^c^	0.20 ± 0.03 ^c^	0.21 ± 0.02 ^c^	0.19 ± 0.01 ^c^	0.20 ± 0.01 ^c^	0.32 ± 0.04 ^b^
	6.204	Ethyl pentanoate	<1100	0.02 ± 0.00	n.d.	n.d.	n.d.	n.d.	n.d.	n.d.
		**∑ SCFAEE**		1.76 ± 0.05 ^a^	0.81 ± 0.05 ^c^	0.77 ± 0.09 ^c^	0.76 ± 0.10 ^c^	0.70 ± 0.10 ^c^	0.66 ± 0.04 ^c^	1.13 ± 0.13 ^b^
**MCFAEEs**	**9.750**	Ethyl hexanoate	1185	0.19 ± 0.02 ^b^	2.36 ± 0.13 ^a^	2.41 ± 0.24 ^a^	2.24 ± 0.05 ^a^	2.07 ± 0.01 ^a^	2.18 ± 0.21 ^a^	2.34 ± 0.24 ^a^
	19.923	Ethyl octanoate	1409	n.d.	23.78 ± 2.03 ^b^	20.46 ± 0.94 ^b^	33.42 ± 3.23 ^ab^	33.32 ± 2.83 ^ab^	35.44 ± 3.22 ^a^	44.55 ± 7.79 ^a^
	31.144	Ethyl decanoate	1591	n.d.	15.78 ± 1.60 ^d^	16.74 ± 0.25 ^cd^	22.50 ± 0.65 ^abc^	18.44 ± 1.03 ^bcd^	24.23 ± 2.63 ^ab^	28.39 ± 3.67 ^a^
	32.928	Ethyl 9-decenoate	1606	n.d.	1.48 ± 0.33 ^n.s.^	1.67 ± 0.07	1.42 ± 0.19	1.12 ± 0.04	1.35 ± 0.14	1.89 ± 0.35
	36.046	Ethyl laurate	1790	n.d.	0.13 ± 0.02 ^de^	0.98 ± 0.23 ^b^	1.62 ± 0.12 ^a^	0.52 ± 0.10 ^c^	0.36 ± 0.03 ^cd^	0.35 ± 0.05 ^cd^
		**∑ MCFAEEs**		0.19 ± 0.02 ^c^	43.52 ± 4.03 ^b^	43.25 ± 1.65 ^b^	61.86 ± 4.20 ^a^	55.70 ± 4.01 ^ab^	64.51 ± 6.19 ^a^	69.23 ± 6.87 ^a^
**LCFAEEs**	39.044	Ethyl tetradecanoate	2007	n.d.	0.73 ± 0.01 ^n.s.^	0.57 ± 0.10	0.69 ± 0.04	0.70 ± 0.09	0.75 ± 0.08	0.72 ± 0.09
	40.829	Ethyl hexadecanoate	2136	n.d.	0.68 ± 0.10 ^b^	0.59 ± 0.02 ^b^	0.85 ± 0.03 ^b^	0.65 ± 0.01 ^b^	0.85 ± 0.08 ^b^	1.39 ± 0.24 ^a^
		**∑ LCFAEEs**		n.d.	1.40 ± 0.11 ^b^	1.16 ± 0.07 ^b^	1.54 ± 0.02 ^b^	1.35 ± 0.08 ^b^	1.60 ± 0.16 ^b^	2.11 ± 0.30 ^a^
**Other Ester**	**5.193**	Isobutyl butyrate	<1100	0.02 ± 0.00	n.d.	n.d.	n.d.	n.d.	n.d.	n.d.
	6.296	Isobutyl butyrate	<1100	0.03 ± 0.00	n.d.	n.d.	n.d.	n.d.	n.d.	n.d.
	28.261	Ethyl 3-acetoxybutyrate	1552	0.03 ± 0.00	n.d.	n.d.	n.d.	n.d.	n.d.	n.d.
	37.298	Hydrocinnamyl isobutyrate	1833	0.02 ± 0.00	n.d.	n.d.	n.d.	n.d.	n.d.	n.d.
		**∑ Other Ester**		0.09 ± 0.01	n.d.	n.d.	n.d.	n.d.	n.d.	n.d.
	2.825	Ethanol	<1100	0.06 ± 0.03 ^b^	59.73 ± 4.65 ^a^	68.43 ± 3.21 ^a^	57.21 ± 2.93 ^a^	62.70 ± 0.63 ^a^	67.91 ± 4.07 ^a^	65.83 ± 12.30 ^a^
**HA**	**5.658**	Isobutyl alcohol	<1100	n.d.	1.56 ± 0.08 ^ab^	1.61 ± 0.17 ^ab^	1.40 ± 0.02 ^b^	1.93 ± 0.18 ^a^	1.56 ± 0.09 ^ab^	1.72 ± 0.29 ^ab^
	9.202	2-Methyl-1-butanol	1168	0.04 ± 0.01	n.d.	n.d.	n.d.	n.d.	n.d.	n.d.
	9.733	Isoamyl alcohol	1185	n.d.	18.70 ± 0.76 ^b^	19.21 ± 0.29 ^ab^	20.21 ± 0.84 ^ab^	22.07 ± 1.62 ^ab^	20.11 ± 0.65 ^ab^	24.17 ± 3.45 ^a^
	19.630	2-Octanol	1404	0.02 ± 0.00 ^a^	n.d.	n.d.	n.d.	n.d.	n.d.	n.d.
	23.385	2-Ethyl-1-hexanol	1475	0.05 ± 0.00	n.d.	n.d.	n.d.	n.d.	n.d.	n.d.
	26.011	2,3-Butanediol	1518	n.d.	1.08 ± 0.04 ^ab^	0.62 ± 0.10 ^bc^	1.38 ± 0.34 ^a^	0.41 ± 0.11 ^cd^	0.42 ± 0.22 ^cd^	n.d.
	32.746	(Z)-3-Nonen-1-ol	1604	0.02 ± 0.00	n.d.	n.d.	n.d.	n.d.	n.d.	n.d.
	33.213	Methionol	1609	n.d.	n.d.	n.d.	n.d.	n.d.	0.32 ± 0.04 ^a^	0.25 ± 0.00 ^b^
	33.598	(6 Z)-Nonen-1-ol	1613	0.03 ± 0.01	n.d.	n.d.	n.d.	n.d.	n.d.	n.d.
	34.218	(E,Z)-3,6-Nonadien-1-ol	1620	0.06 ± 0.01 ^c^	0.12 ± 0.01 ^ab^	0.12 ± 0.00 ^ab^	0.12 ± 0.02 ^ab^	0.11 ± 0.00 ^bc^	0.10 ± 0.02 ^bc^	0.17 ± 0.04 ^a^
	36.150	Benzyl alcohol	1794	0.01 ± 0.00 ^c^	n.d.	n.d.	n.d.	0.98 ± 0.14 ^a^	0.18 ± 0.01 ^b^	0.16 ± 0.02 ^bc^
	36.696	2-Phenylethanol	1813	0.02 ± 0.00 ^d^	4.71 ± 0.24 ^c^	5.46 ± 0.44 ^bc^	6.45 ± 0.48 ^b^	5.75 ± 0.53 ^bc^	6.80 ± 0.30 ^ab^	8.16 ± 0.96 ^a^
	41.030	2,4-Di-tert-butylphenol	2213	0.01 ± 0.00 ^c^	1.75 ± 0.04 ^b^	1.99 ± 0.08 ^ab^	1.94 ± 0.02 ^ab^	2.35 ± 0.27 ^a^	1.88 ± 0.12 ^ab^	2.28 ± 0.29 ^a^
		**∑ HA**		0.27 ± 0.03 ^c^	27.92 ± 0.98 ^b^	29.01 ± 0.57 ^ab^	31.51 ± 1.66 ^ab^	33.60 ± 2.73 ^ab^	31.38 ± 0.44 ^ab^	36.92 ± 4.88 ^a^
**Other volatiles**	**10.207**	Styrene	1199	n.d.	n.d.	n.d.	0.27 ± 0.03 ^n.s.^	0.27 ± 0.03	0.22 ± 0.01	0.29 ± 0.06
	19.291	1,3-Di-tert-butylbenzene	1392	0.02 ± 0.01 ^c^	0.67 ± 0.06 ^b^	0.64 ± 0.03 ^b^	0.88 ± 0.08 ^ab^	1.04 ± 0.24 ^a^	0.85 ± 0.05 ^ab^	0.86 ± 0.07 ^ab^
	34.810	Oxime-methoxy phenyl	1803	0.02 ± 0.00 ^c^	0.47 ± 0.04 ^b^	0.56 ± 0.04 ^ab^	0.60 ± 0.04 ^ab^	0.69 ± 0.06 ^a^	0.69 ± 0.07 ^a^	0.76 ± 0.13 ^a^
	38.577	Octanoic acid	1876	n.d.	1.87 ± 0.36 ^b^	1.97 ± 0.28 ^b^	2.06 ± 0.18 ^b^	1.54 ± 0.12 ^b^	2.01 ± 0.25 ^b^	3.02 ± 0.53 ^a^

Mean (*n* = 3 ± SE). RT: Retention time. KI: Kovats index. n.d.: not detected. Different letters in the same row indicate significant differences (*p* < 0.05) among must, still wine, and sparkling process. AHA: Acetate higher alcohol. SCFAEEs: Short-chain fatty acid ethyl ester. MCFAEEs: Medium-chain fatty acid ethyl ester. LCFAEEs: Long-chain fatty acid ethyl ester. HA: Higher alcohols. ns: statistically non-significant differences.

**Table 7:** Evolution of principal odor activity value (OAV > 1) in enriched must, still wine, and during the sparkling process of melon-based wine (10 weeks).

Compounds	OTH	Must	Still Wine	Sparkling Wine (Weeks)					Odor Descriptor
				2	4	6	8	10	
**AHA**									
Isobutyl acetate	1.605	<1	2.2 ± 0.1 ^b^	3.0 ± 0.4 ^b^	2.9 ± 0.4 ^b^	4.2 ± 0.9 ^a^	2.6 ± 0.2 ^b^	2.4 ± 0.3 ^b^	Sweet, fruity, banana, tropical
Isoamyl acetate	0.03	17.7 ± 1.1 ^c^	480.9 ± 11.8 ^b^	495.9 ± 2.1 ^b^	512.9 ± 34.2 ^b^	483.7 ± 39.7 ^b^	503.4 ± 32.1 ^b^	646.8 ± 93.2 ^a^	Sweet banana, and fruity
3,6-Nonadienyl acetate	0.015	<1	83.1 ± 0.5 ^bc^	59.2 ± 26.2 ^c^	108.2 ± 17.4 ^abc^	72.3 ± 22.4 ^c^	132.1 ± 30.6 ^ab^	121.7 ± 18.3 ^a^	Fruity
2-Phenylethyl acetate	0.25	<1	15.4 ± 1.3 ^b^	16.4 ± 1.0 ^b^	16.3 ± 1.3 ^b^	17.0 ± 1.8 ^b^	16.1 ± 0.6 ^b^	20.5 ± 2.4 ^a^	Roses and honey aroma
**SCFAEE**									
Ethyl butanoate	0.6	1.1 ± 0.1 ^ab^	1.1 ± 0.1 ^b^	<1	<1	<1	<1	1.4 ± 0.2 ^a^	Fruity, sweet, tutti frutti, apple
Ethyl 2-methylbutyrate	0.074	10.5 ± 0.9 ^a^	2.3 ± 0.2 ^c^	2.7 ± 0.4 ^bc^	2.8 ± 0.3 ^bc^	2.6 ± 0.2 ^c^	2.7 ± 0.1 ^bc^	4.3 ± 0.6 ^b^	Sharp, sweet, green apple, fruity
**MCFAEE**									
Ethyl hexanoate	0.1	1.9 ± 0.2 ^b^	23.6 ± 1.3 ^a^	24.1 ± 2.4 ^a^	22.4 ± 0.5 ^a^	20.7 ± 0.1 ^a^	21.8 ± 2.1 ^a^	23.4 ± 2.4 ^a^	Apple peel fruit
Ethyl octanoate	0.6	<1	39.6 ± 3.4 ^cd^	34.1 ± 1.6 ^d^	55.7 ± 5.4 ^bc^	55.5 ± 4.7 ^bc^	59.1 ± 5.4 ^b^	74.3 ± 13.0 ^a^	Pineapple, pear, soapy
Ethyl decanoate	0.2	<1	78.9 ± 8.0 ^d^	83.7 ± 1.3 ^d^	112.5 ± 3.2 ^bc^	92.2 ± 5.2 ^cd^	121.1 ± 13.2 ^b^	141.9 ± 18.3 ^a^	Sweet, fruity, nuts, dried fruit
Ethyl 9-decenoate	0.1	<1	14.8 ± 3.3 ^ab^	16.7 ± 0.7 ^ab^	14.2 ± 1.9 ^b^	11.2 ± 0.4 ^b^	13.5 ± 1.4 ^b^	18.9 ± 3.5 ^a^	Floral
Ethyl laurate	0.5	<1	<1	2.0 ± 0.5 ^b^	3.2 ± 0.2 ^a^	1.0 ± 0.2 ^c^	<1	<1	Sweet
**LCFAEE**									
Ethyl tetradecanoate	0.5	<1	1.5 ± 0.0 ^b^	1.1 ± 0.2 ^bc^	1.4 ± 0.1 ^bc^	1.4 ± 0.2 ^bc^	1.5 ± 0.2 ^a^	1.4 ± 0.2 ^bc^	Sweet fruit, butter, fatty odor
Ethyl hexadecanoate	1	<1	<1	<1	<1	<1	<1	1.4 ± 0.2	Fruity, concord grape
**HA**									
(6Z)-Nonen-1-ol	0.001	32.2 ± 5.3	<1	<1	<1	<1	<1	<1	Sweet, fatty, melon-like
(E,Z)-3,6-Nonadien-1-ol	0.003	20.2 ± 2.0 ^c^	41.6 ± 2.9 ^b^	41.3 ± 0.2 ^b^	41.6 ± 5.2 ^b^	36.9 ± 0.7 ^bc^	33.6 ± 6.9 ^bc^	56.4 ± 14.5 ^a^	Melon-like, leafy, nutty odor

Mean (*n* = 3 ± SE). Different letters in the same row indicate significant differences (*p* < 0.05) among must, still wine, and sparkling process. OAV value < 1. OTH: Odor threshold.

**Table 8:** Sensory evaluation of the melon sparkling wine at 10 weeks of storage.

Organoleptical Characteristics	Sparkling Melon-Wine	Range (Excellent to Inadequate)
**Visual**	**14.44 ^z^ ± 0.24**	**(15–3)**
Limpidity	4.67 ± 0.17	(5–1)
Aspect other than limpidity	9.78 ± 0.22	(10–2)
**Nose**	**28.67 ± 0.69**	**(30–12)**
Genuineness	5.78 ± 0.15	(6–2)
Positive intensity	7.78 ± 0.15	(8–2)
Quality	15.11 ± 0.48	(16–8)
**Taste**	**39.11 ± 0.99**	**(44–18)**
Genuineness	5.44 ± 0.18	(6–2)
Positive intensity	7.11 ± 0.11	(8–2)
Harmonious persistence	7.22 ± 0.22	(8–4)
Quality	19.33 ± 0.60	(22–10)
**Harmony-Overall judgement**	**9.89 ± 0.20**	**(11–7)**
**TOTAL**	**92.11 ± 1.77**	**(100–40)**

^z^ Mean (*n* = 14 ± SE).

## Data Availability

The datasets supporting the findings of this article are available upon reasonable request.
